# Heart Rate Variability Changes in Business Process Outsourcing Employees Working in Shifts

**Published:** 2010-10-31

**Authors:** Kirthana U Kunikullaya, Suresh K Kirthi, D Venkatesh, Jaisri Goturu

**Affiliations:** 1 Department of Physiology, M S Ramaiah Medical College, Bangalore, India - 560 054; 2 R & D Services, MindTree limited, Global Village, RVCE Post, Bangalore, India - 560 059

**Keywords:** Heart rate variability, autonomic functions, business process outsourcing, shift work

## Abstract

**Background and Objectives:**

Irregular and poor quality sleep is common in business process outsourcing (BPO) employees due to continuous shift working. The influence of this on the cardiac autonomic activity was investigated by the spectral analysis of heart rate variability (HRV).

**Methods:**

36 night shift BPO employees (working from 22:00 to 06:00h) and 36 age and sex matched day shift BPO employees (working from 08:00 to 16:00h) were recruited for the study. Five minute electrocardiogram (ECG) was recorded in all the subjects. Heart rate variability was analyzed by fast Fourier transformation using RMS Vagus HRV software. The results were analyzed using Mann Whitney U test, Student t-test, Wilcoxon signed rank test and were expressed as mean ± SD.

**Results:**

Sleepiness was significantly higher among night shift workers as measured by Epworth Sleepiness Scale (p<0.001). Night shift BPO employees were found to have a trend towards lower values of vagal parameters - HF power (ms^2^), and higher values of sympathovagal parameters like LF Power (ms^2^) and the LF/HF power (%) suggesting decreased vagal activity and sympathetic over activity, when compared to day shift employees. However, HRV parameters did not vary significantly between the day shift employees and night shift workers baseline values, and also within the night shift group.

**Interpretation and Conclusion:**

Night shift working increased the heart rate and shifted the sympathovagal balance towards sympathetic dominance and decreased vagal parameters of HRV. This is an indicator of unfavorable change in the myocardial system, and thus shows increased risk of cardiovascular disease among the night shift employees.

## Introduction

The cardiovascular disease (CVD) burden is rising in developing countries like India. Additionally, CVD in Indians has been shown to occur at least a decade or two earlier than their counterparts in developed countries [1]. Olsen and Kristensen estimated that, the attributable risk of work environment on chances of developing CVD to be 20%, that is, that 1/5 of CVD cases would not exist if all work environment risk factors were entirely removed [2]. In many occupational settings, such as BPO employees (business process outsourcing in telephonic related voice support or call centers) who work during non - conventional hours suffer from sleep problems due to disruptions in their normal sleep cycle. These industries must operate 24 hours per day because the production process is much longer than 8 hours and must be performed continuously. Shift work results in misalignment between the circadian timing and the sleep - wake cycle, leading to irregular and poor quality sleep [3].  Recent studies also indicate that sleep deprivation due to shift working may affect the cardiac autonomic nervous system (ANS), contributing to high cardiovascular risk. However, results from individual studies have not been wholly consistent [4]. In the last two decades a significant correlation has been established between altered autonomic function and cardiovascular mortality, including sudden cardiac death. Heart rate variability (HRV) represents one of the most reliable ANS markers. Heart rate and its variability are under sympatho - vagal influence, and reduced heart rate variability and increased heart rate are the result of autonomic imbalance [5].  In normal population, autonomic activity shows a circadian pattern, with sympathetic activity predominant during the day and parasympathetic activity at night [6].

Effects of sleep deprivation on neural cardiovascular control may have important clinical implications. However, the mechanisms underlying this elevated risk remain unclear. It has been suggested that fragmented sleep or sleep deprivation may increase the incidence of CVD. Several studies have proposed the hypothesis that activation of the sympathetic system by sleep deprivation may be implicated in triggering cardiovascular events in the morning hours. Insomnia is associated with increased heart rate, low frequency spectral power, an indicator of increased sympathetic system activity and decreased high frequency power which imply that, chronic insomniacs could be at increased risk of development of CVD [7].  In the present study we have tried to analyze the change in HRV in night shift workers due to the cumulative sleep deprivation following one week of continuous night shift duty and also investigate the effect of night shift work on the cardiac status of the shift worker, compared to day shift worker so that any complication arising of it can be detected early.

## Materials and Methods

This study was conducted by the Department of Physiology, M. S. Ramaiah Medical College (MSRMC), Bangalore, Karnataka. The study protocol followed was in accordance with the ethical standards of the committee on human experimentation of the institution and was approved by the local ethical clearance committee of the institution. Written informed consent was obtained from each subject separately, after explaining the protocol of the study in their local language. The subjects were categorized as healthy after a detailed history taking and relevant clinical examination. The inclusion criteria were as follows: All the individuals working in BPO companies in shifts were included in the study. Both day and night shift workers doing similar kind of work, with similar stress. The employees working for more than 2 months in the company, aged between 18 to 55 years, of either gender, working on telephonic or voice support were recruited as subjects. Thirty six night shift BPO employees (subjects) were compared with age and sex matched 36 day shift workers (normal sleepers). The exclusion criteria were: Individuals with other pre-existing endocrinal diseases, cardiac problems, psychiatric disorders, medical problems (non - diabetic, non - hypertensive); volunteers who are on any drugs like amphetamines, sedatives, benzodiazepines, central nervous system stimulants, steroids or any other drug that grossly affects the sleep; people who were smokers; day shift employees in particular, who have not done night shift in their lifetime.

The night shift call center employees included in the study were on medium rotating night shift schedule as quoted by Amelsvoort et al [8]. According to this, these employees were working 7 consecutive night shifts and then were given a day off, followed by 14 days of day shift before the next night shift began. The night shift started from 22:00 to 06:00 IST (8 hrs/day or 56 hrs/week). However, the day shift workers included in the study were working from 08:00 to 16:00 (8hrs/day) in a different BPO company. Both the companies chosen for the study were doing similar work, so that the work stress between day and night shift employees matched. Subjects were made to fill a standard questionnaire which enquired about their general health, menstrual history, drugs and previous hospitalization. A sleep questionnaire of Epworth Sleepiness Scale (ESS) was administered to all the participants [9].

HRV apparatus was set up in a room in both the companies. The room was well ventilated, with no external disturbances. Subject was made to lie down and relax in supine position for 15 minutes before the procedure. Electrocardiogram (ECG) in lead II was recorded for 5 minutes on all the subjects for baseline values under resting condition. The baseline data for the study group (the night shift workers) was recorded during the first 2 hours of commencement of work. The HRV for the sleep deprived group was recorded during the last 2 hours of their duty time on the last day of the night shift. Baseline recording was taken between 22:00 to midnight on the first day and follow up recording was taken between 04:00 to 06:00. Thus among night shift employees ECG recording was done the second time at the end of one week of continuous night duty. The control values were also recorded in the day shift workers during the first 2 hours of their working. HRV was measured using RMS Vagus HRV software (RMS, India). Power spectral density was calculated by fast Fourier transform for 5 minutes. Low frequency power (LF) component and high frequency power (HF) component were defined as the power at 0.04-0.15 and 0.15-0.4 Hz, respectively [10]. The LF and HF components were expressed as absolute values (ms^2^), power (%) and normalized values (n.u). SPSS 15.0, Stata 8.0, MedCalc 9.0.1 and Systat 11.0 were used for the analysis of the data.  For all the parameters, mean ± SD were calculated. The measured HRV was compared between night shift and day shift call center employees.

### Statistical Analysis

The measured HRV was compared between night shift and day shift call center employees. All the parameters were expressed as mean ± SD. SPSS 15.0, Stata 8.0, MedCalc 9.0.1 and Systat 11.0 were used for the analysis of the data. Descriptive statistical analysis was done.

a) Student t test (two tailed, independent) was used to find the significance of study parameters on continuous scale between two groups (Inter group analysis) and Mann Whitney U test was used to find the significance of parameters in non-parametric condition.

b) Student t test (two tailed, dependent) was used to find the significance of study parameters on continuous scale within each group.

c) Wilcoxon signed rank test was used to find the significance (with in group) between baseline values and values at the end of one week of night shift.

d) Student t test (two tailed; independent) was used to test the homogeneity of samples based on age (or continuous parameters) and Chi-square test to test the homogeneity of samples based on parameters on categorical scale between two groups. Significance was assessed at 5 % level (p < 0.05).

## Results

Sleepiness was significantly higher among night shift workers as measured by ESS (p<0.001). Mean RR interval was lower and mean heart rate was higher in night shift workers than the day shift workers though not significant. HRV parameters were not significantly different in the two groups (Table 1). Mean RR interval was significantly lower (p<0.05) and mean heart rate was significantly higher (p<0.05) in night shift workers after one week of continuous night shift showing a shift towards sympathetic dominance in the same. None of the HRV parameters showed any significant change within night shift workers group (Table 2). Mean RR interval decreased and mean HR increased significantly (p<0.001) on comparison between the day shift and night shift workers after one week of night duty. However, HRV parameters showed no significant difference, though a trend towards decreased parasympathetic response {HF (ms^2^)} and increased sympathovagal balance as seen by LF/HF power%, LF power (ms^2^) (Table 3).

## Discussion

Autonomic nervous control of the cardiovascular system has a distinct circadian rhythm, and this may be an important mechanism underlying the diurnal distribution of cardiac events such as myocardial infarction and sudden cardiac death [11]. A non-invasive technique used for investigating cardiovascular autonomic control is the analysis of HRV in the frequency domain [12]. Decrease of HRV is frequently associated with coronary artery disease (CAD), and the degree of this impairment is reported to be a predictor of mortality in such patients [13]. It is generally considered that the absolute values of HF component are markers for vagal activity, whereas LF or LF/HF reflects the sympathetic activity or sympatho-vagal balance [10].

Shift workers have been shown to experience reduced concentration, attention span and increased reaction time as well as gastrointestinal problems and increased risk for heart attacks [14]. Specifically, night work is problematic because shift workers are working when the underlying circadian rhythm is performing at its lowest [15]. Further, problems arise because shift workers attempt to sleep during the day when the body is supposed to be active  [16]. As a result, day sleep following a night of sleep deprivation is typically of poorer quality and shorter duration when compared to regular night sleep, even under optimal conditions [17]. Due to frequent sleep disruption, cumulative sleep loss is a salient problem among shift workers performing night duties regularly [18]. The present study has shown that the night shift workers have a significantly increased sleepiness (p<0.001) as measured by subjective scoring - the Epworth sleepiness scale. Sleep restriction over the course of six nights resulted in increased sympathetic activity as assessed by HRV analysis [19].

The hazard of night work was recognized by the father of occupational medicine, Bernardini Ramazzini (1633-1714) [20]. Several studies have reported a higher prevalence of coronary risk factors among shift workers which include increased smoking, higher blood pressure, increased serum cholesterol, glucose, uric acid levels and urinary adrenaline excretion [21]. One of the consequences of shift work is that it produces maladjustment of circadian rhythm known as internal desynchronization. However, the way in which the work schedule affects the heart is not clear. One of the factors in causation of heart disease may be disturbed cardiac autonomic control.

In this study night shift BPO employees, were found to have lower values of vagal parameter - HF power (ms^2^), and higher values sympathovagal parameters LF Power (ms2) and LF/HF Power (%), suggesting reduced cardiac parasympathetic activity and increased sympathetic activity, compared with the day shift employees.

Xu Zhong et al, showed that HF (ms^2^) was decreased LF/HF power % was increased after sleep deprivation. They concluded that acute sleep deprivation was associated with increased sympathetic and decreased parasympathetic cardiovascular modulation [22]. Tochikubo et al, suggested that fatigue and mental stress due to lack of sleep may influence sympathovagal balance [23]. Ludovic et al, showed that the type of shift schedule was a significant modifier of HRV change in shift workers [8]. However, some studies like those by Furlan et al, showed a lower sympathetic activity in shift workers. They showed that reduced values may be related to the presence of sleepiness or diminished alertness, which in turn facilitate errors and accidents [24]. HRV change was not significant even in other studies like Kastanioti et al [25], Freitas et al [26], Thomas et al [27].

Electrocardiogram (ECG) recording was done only for 5 minutes in this study. If a twenty four hour ECG had been done, the results might have been more conclusive. However, a five minute ECG HRV analysis can be used as a predictor of disturbance in autonomic function [10]. The present study has been done on employees working in BPO companies, whose population has increased significatly in India in the recent past. Most of other studies have been done on shift workers like truck drivers, nurses and doctors. The BPO employees have maximum sleep deprivation as they are monitored and work under tremendous stress unlike the other groups. The stress is compounded by abusive, rude customers, poor working facilities, inadequate monetary compensation, strict monitoring by the management and repetitive nature of job  [28]. There is an urgent need to address the health related issues of BPO employees to improve the health status and productivity of the industry [29].

From the above discussion, we observe that there is a trend towards increased sympathetic activity as shown by LF/HF power (%) and LF power (ms^2^) among shift workers performing night duty regularly over a period of years. However, the results were not significant at p< 0.05. Sympathetic over activity may play a role in the excessive rate of cardiovascular disease described in habitual shift workers. Further studies are needed to establish conclusively that, HRV arising out of chronic sleep deprivation is a prominent risk factor for CAD.

## Figures and Tables

**Table 1:**
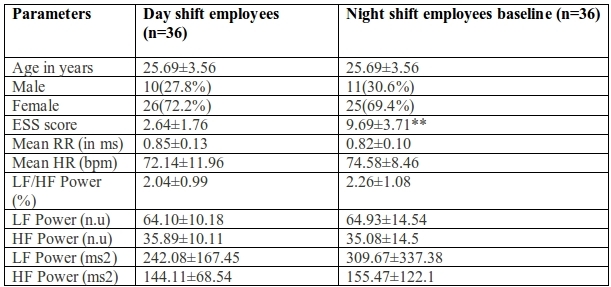
Comparison of baseline parameters between day shift & night shift employees

*p< 0.05, **p< 0.01; ESS: Epworth sleepiness scale; LF/HF Power (%): Ratio LF [ms^2^]/HF[ms^2^]; n.u: normalized units; Powernu = (100 X absolute power) / (total power - VLF); bpm: Beats per minute; ms: Milliseconds.

**Table 2:**
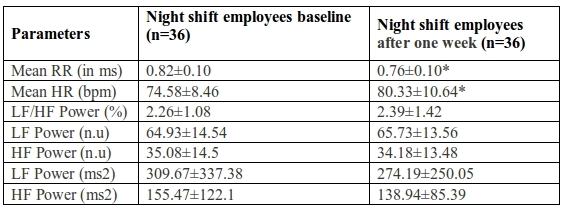
Comparison of baseline parameters of night shift employees with parameters after one week of night duty

**Table 3:**
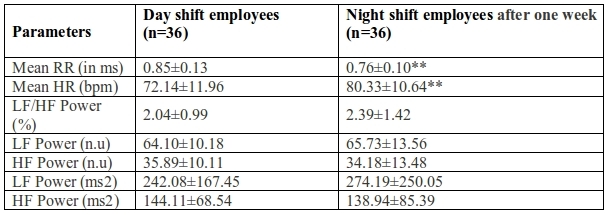
Comparison of parameters in day shift & night shift employees after one week of night duty
